# EEG‐biomarker theta/beta ratio and attentional quotients in adults who stutter: An electrophysiological and behavioral study

**DOI:** 10.1002/brb3.2812

**Published:** 2022-12-02

**Authors:** Ahmad Poormohammad, Ali Mohammad Pourrahimi, Mazyar Fathi, Sara Sardari, Mahdiye Sarrafe Razavi, Mohammad Karimi Bahrasemani, Anusheh Mosanen Mozaffary, Shahrzad Mazhari

**Affiliations:** ^1^ Neuroscience Research Center, Institute of Neuropharmacology Kerman University of Medical Sciences Kerman Iran; ^2^ Division of Neurocognitive Sciences, Psychiatry and Behavioral Sciences Research Center Mashhad University of Medical Sciences Mashhad Iran; ^3^ Novin Rehabilitation Clinic Kerman Iran; ^4^ Department of Speech Therapy, Ibn‐e‐Sina Psychiatric Hospital Mashhad University of Medical Sciences Mashhad Iran; ^5^ Department of Psychiatry, Medical School Kerman University of Medical Sciences Kerman Iran

**Keywords:** attention, AWS, IVA, TBR

## Abstract

**Introduction:**

There is increasing evidence that connects developmental stuttering to attention. However, findings have represented contradiction. Therefore, this study was conducted to investigate the possible relationship between stuttering and attention in resting and undertask conditions.

**Methods:**

In a cross‐sectional study, 26 right‐handed AWS (adults who stutter) and 25 matched fluent speakers were enrolled. Demographic data were collected, and the Beck anxiety inventory (BAI) was filled out for all participants. Then, QEEG was conducted, followed by IVA2. CPT test for all subjects. Finally, data were analyzed using SPSS software version 16.

**Results:**

AWS indicated significantly weaker auditory focus attention in the task (*p* = .02) than the control group, while a similar resting‐state EEG marker of attention was found between groups (*p* > .05). Moreover, attention was not correlated between the two conditions (*p* > .05).

**Conclusion:**

The EEG marker of attention did not necessarily designate the attentional performance of AWS under the task. Furthermore, attentional skills could be considered in the assessment and therapeutic programs of at least some groups of AWS.

## 1 INTRODUCTION

Stuttering is a neurodevelopmental disorder that affects speech fluency, begins in childhood, and persists in 1% of adults (Eton & Lepore, 2008). It is described by speech motor behavior disruptions resulting in sound and syllable repetitions, sound prolongations, and broken words. Stuttering negatively influences the quality of life and vocational and emotional consequences (Yaruss & Quesal, 2006). Although neuroimaging, lesion, pharmacological, and genetic studies have described different neural circuitries implicated in stuttering, the etiology is still unknown (Craig‐McQuaide et al., 2014). Some scientists believe that atypical attentional processing may participate in stuttering (Chanpimol et al., 2017; Piispala et al., 2016). This problem in attentional processing may lead to slower stimulus evaluation and therefore prolonged response selection process and may disturb the execution process and motor sequences (Piispala et al., 2016). This hypothesis follows poorer motor learning skills in stuttering (Chanpimol et al., 2017). Although it is highly believed that the development of stuttering is related to complicated interactions among linguistic, emotional, cognitive, and motor factors, the contributions of attentional control have not been well understood. It is said that children's risk for persistent stuttering may increase if they cannot shift their attention and move through the experience of stuttering (Eichorn et al., 2018).

In addition to other critical functions like cognitive control, attention is part of other momentous skills named executive functions (EFs) (Hildebrandt, 2018; Miyake et al., 2000). These components are interdependent, difficult to separate, and necessary for higher‐order skills like reasoning, problem‐solving, and planning. While several studies have compared these skills in children and adults who stutter and their counterparts, the findings are still controversial. For example, in a GO/Nogo study, CWS showed longer latencies in N2 and P3 components of Go trials but did not differ in Nogo trials from typically developed children. They also acted similarly to the control group behaviorally. In the final analysis, the authors did not confirm less efficacious response inhibition in CWS but suggested atypical attentional processing (Piispala et al., 2016). However, in another study, in a GO/Nogo task, CWS showed atypical response inhibition processes compared with fluent counterparts (Piispala et al., 2017). Moreover, in a meta‐analysis study, both inhibitory and attentional skills, which parents rated, obtained lower scores in CWS than fluent speakers, while the two groups acted similarly in equivalent behavioral measures (Ofoe et al., 2018). AWS's contradictions become even more complex due to the chronicity of symptoms and negative communication experiences. In a meta‐analysis study, adults who stutter (AWS) demonstrated weaker attentional function than the control group. However, the association of stuttering with attentional abilities was not observed in all subjects, so the results were not confirmatory and definite (Doneva, 2020). One of the reasons for these controversies comes back to the complex and multifactorial nature of attention. It is believed that two interconnected systems regulate attentional control; the bottom‐up system that detects stimuli by structures like AC, thalamus, and amygdala, and the top‐down system that maintains attention to relevant tasks by higher‐level cortical structures like DLPFC. Some findings support the idea that the theta/beta ratio or TBR (EEG marker of attention) is an index of this top‐down control (Angelidis et al., 2016). Interestingly, this index was found to have a negative correlation with the behavioral aspect of attention (Kim et al., 2015; Putman et al., 2010; Putman et al., 2014). This negative correlation may be caused by the fact that a higher amplitude of theta wave is concerned with lower glucose metabolism and, consequently decline in attention and other brain performances (Zametkin et al., 1993). So, for better evaluation of attention, assessment of both physiological and behavioral aspects is preferable. For behavioral assessment of attention, Integrated Visual and Auditory Continuous Performance Test (IVA + CPT) seems to be a comprehensive behavioral test that covers most aspects of the attentional and inhibitory performance in both visual and auditory modalities simultaneously. This test is based on data collected from 1700 normally developed men and women aged 6−96 years (Kim et al., 2015), designed to diagnose ADHD. As performing the test requires a transition of attention between the visual and auditory systems simultaneously, split attention, which is highly correlated with the prefrontal lobe function, is triggered in this test (Loose et al., 2003). This brain area is assumed to play an essential part in the pathophysiology of stuttering (Craig‐McQuaide et al., 2014; Xuan et al., 2012). As AWS reports significant ADHD‐like behaviors in their childhood history (Alm & Risberg, 2007) and shows an ADHD‐type EEG profile (Ratcliff‐Baird, 2002), IVAcpt2 is expected to be a suitable test for examining AWS's attentional performance. The validity of this test was proved in a study in which the correlation between IVA.CPT, Working Memory, and Processing Speed Indexes of the Wechsler Intelligence Scales (WISC‐IV), and parents' and teachers' ratings of child behaviors were assessed (Arble et al., 2014). This test provides a total of 12 quotients with separate visual and auditory scores, which are reported automatically after completion of the test. The quotients involve response control primary scales, including prudence (errors of commission), consistency (general reliability of reaction times), stamina (reaction time for trials toward the end of the test compared with reaction time for early trials), and primary attention scales, including vigilance (errors of omission), focus (variation of processing speed), and speed (mean reaction time for correct responses) (Arble et al., 2014). Considering the above, we predict that TBR as a probable index of top‐down attentional processing might correlate with IVA quotients negatively.

After all, the present study aimed to explore if facets of attention differ between AWS and their fluent counterparts and any relationship between them. As the problem in attentional processing at the physiological level may affect response selection and outcome, it is expected that TBR shows a negative relation with behavioral measures of IVA2.CPT. For the first time, this pilot study compared both the resting‐state (electrophysiological) and the undertask (by a rule‐switch task) aspect of attention between AWS and the control group by TBR and IVA2.CPT, respectively, to provide some evidence about the role of attention in stuttering and smooth the way for more comprehensive and detailed upcoming studies.

## 2 METHOD

### 2.1 Participants

Twenty‐six right‐handed AWS that referred to the speech therapy clinics and 25 fluent speakers, matched in age, sex, handedness, and education, participated in this cross‐sectional study. The study size arrived by considering previous similar studies (Bayat et al., 2020; Chanpimol et al., 2017; Ratcliff‐Baird, 2002). All subjects had normal or corrected‐to‐normal vision and audition, and none had a psychiatric or neurological disease history. After signing the consent form, the researcher explained how to perform the tests. A Beck anxiety inventory (BAI) was also filled out to assess anxiety (Beck et al., 1988). Then, QEEG was conducted, followed by IVA2. CPT test.

### 2.2 Analyzing the speech samples

Five minutes of spontaneous speech of AWS and 5 min of reading a standard passage were recorded and analyzed. The average of the three most prolonged duration of stuttering occurrences was calculated for stuttered syllables. The severity of associated behaviors, often seen with stuttering, was numbered according to the Likert‐five scale judgment. The final scores were calculated according to SSI_4_. It is a standard test for assessing the severity of stuttering according to the three items: (i) the number of disfluencies, (ii) averaged duration of speech disfluencies, and (iii) associated behaviors (Zolfaghari et al., 2014).

### 2.3 BAI

Anxiety is defined as feelings of tension and fear, is often relevant to the anticipation of events, and is accompanied by activation of the autonomic nervous system (Oh et al., 2018). The Beck Anxiety Inventory (BAI) is a widely used 21‐item self‐report inventory consisting of 21 items rated on a 4‐point Likert scale from 0 (not at all) to 3 (severely) used to assess anxiety levels in adults and adolescents (Oh et al., 2018). It proved highly internal consistency (Cronbach's alpha = .94) and acceptable reliability (Fydrich et al., 1992).

### 2.4 QEEG

#### 2.4.1 QEEG recording

The participants were seated in a soundproofed, dimly lit chamber on an armchair, and their heads were fixed on a chin rest to reduce unwanted artifacts. Electroencephalography (EEG) was recorded by a 19‐channel Win EEG system (version 2.126.97, Mitsar Inc, Russia) in eye‐closed condition for 4 min. The sampling rate was 250 Hz, and electrodes were positioned according to the international 10–20 system. Impedance was kept below 5 kΩ. Low‐ and high‐pass filters were 0.1 and 45 Hz, respectively, and the 60‐Hz notch filter was applied. EEG data were recorded using monopolar montage, and input signals were referenced to the linked ear.

#### 2.4.2 QEEG analysis

First, an independent component analysis (ICA) was performed to isolate artifacts caused by eye, muscle movement, and heart noise. Then, two EEG experts visually inspected and corrected EEGs. Finally, 2 min of artifact‐free EEG recordings were selected and imported to Neuroguide software version 1.0.1.0 for later analysis. The recording was analyzed by Fourier transform mathematical processes (FFT) for quantitative analysis. Different bands’ measures were calculated by Neuroguide software, considering age and sex. We were primarily interested in EEG signals of the frontal areas as they are suspicious areas presumed to participate in the pathophysiology of stuttering (Jäncke et al., 2004; Joos et al., 2014). So, we selected the electrodes FP1, FP2, F3, F4, Fz, C3, C4, and Cz. As the final measurements of TBRs were extracted by neuroguide software, we used the default setting for analysis. The theta and beta band frequencies were 4–8_HZ_ and 12–25_HZ_, respectively, and the epoch duration was 4 seconds. The electrodes were considered independent in our analysis.

### 2.5 IVA2.CPT test

The IVA2.CPT test was completed in a soundproof chamber. Subjects were seated in front of the VGA computer monitor about 40–50 cm away from the screen. The left button of an ergonomic mouse was used to record responses. The subject's arm was allowed to rest on an armchair in a comfortable position. The visual stimuli (1 or 2) were in black and were presented for 167 ms inside a rectangle positioned in the center of the screen. The auditory stimuli (1 or 2) lasted for 500 ms. The responses were converted to standard scores (*M* = 100 and SD = 15) based on the normative data from the IVA. A description of instructions for the IVA was played before the warm‐up section. Subjects were asked to click the mouse when they saw or heard a “1” (target) and not click the mouse when they saw or heard a “2.” Ten trials were presented in the warm‐up, and five blocks of 100 trials were presented in the central part. After the 500 trials, the cool‐down section was given, similar to the warm‐up section. The entire test lasted about 20 min to be completed. Measures of the quotients were calculated automatically by software in the report tab.

### 2.6 Statistical analysis

Mann–Whitney *U* test was used for comparing anxiety scores between AWS and the control group. Adjusted by anxiety scores as a covariate, the univariate general linear model was performed for IVA2.CPT quotients and TBR measures to compare scores between AWS and control groups. Pearson's correlation coefficients were applied to calculate the correlation between IVA quotients and TBR scores. The analyses were performed using Statistical Package for the Social Sciences software, version 16, and a *p* value less than .05 was considered significant.

### 2.7 Ethics

This study was approved by the ethics committee of Kerman University of Medical Sciences, Kerman, Iran, with the ethical approval number: IR.KMU.REC.1399.195. All participants signed the consent form.

## 3 RESULTS

### 3.1 Demographic and clinical characteristics

The demographic and clinical characteristics of the study population have been summarized in Table 1. The mean age of cases and controls was 27 ± 6.10 and 26 ± 6.04, respectively, without a significant difference between groups (*p* = .53). However, anxiety had significantly higher scores in the AWS group than in the controls (*p* = .02). Therefore, later analyses were adjusted for anxiety.

**TABLE 1:** Demographic and clinical characteristics of the study participants

Characteristics	AWS	Control	*p* Value
Age	27 ± 6.10	26 ± 6.04	.53
Gender *N* (%male)	18 (64%)	16 (57%)	.26
Education (years)	15.15 ± 3.10	16.88 ± 4	.09
Anxiety	16 ± 8.17	10.48 ± 9.37	.02*

*Note*. Data were presented as mean ± SD.

Abbreviation: AWS: adult who stutter.

* *p* < .05.

### 3.2 Assessment of stuttering severities

According to the SSI4, from the 28 AWSs, 29% were categorized as very mild stutterers, 39% as mild, 14% as moderate, and 18% as severe. Total scores and stuttering severity were reported in Table 2.

**TABLE 2:** Total scores and stuttering severity according to SSI4 in AWS

AWS	Age	Sex	Total score	Severity
1	26	M	11.00	Very mild
2	29	M	33.00	Severe
3	28	F	24.00	Mild
4	31	M	10.00	Very mild
5	30	F	14.00	Very mild
6	17	M	17.00	Very mild
7	21	M	22.00	Mild
8	20	M	20.00	Mild
9	18	M	16.00	Very mild
10	28	F	23.00	Mild
11	28	M	16.00	Very mild
12	36	M	35.00	Severe
13	23	F	25.00	Moderate
14	26	F	21.00	Mild
15	27	M	26.00	Moderate
16	19	M	34.00	Severe
17	28	M	24.00	Mild
18	22	M	19.00	Mild
19	18	M	26.00	Moderate
20	33	M	34.00	Severe
21	27	M	24.00	Mild
22	24	M	19.00	Mild
23	29	M	24.00	Mild
24	37	M	17.00	Very mild
25	18	M	15.00	Very mild
26	30	M	32.00	Severe
27	22	F	19.00	Mild
28	33	M	28.00	Moderate

Abbreviations: AWS: adults who stutter, M: male, F: female.

### 3.3 Comparison between groups: QEEG and IVA.2

**FIGURE 1:**
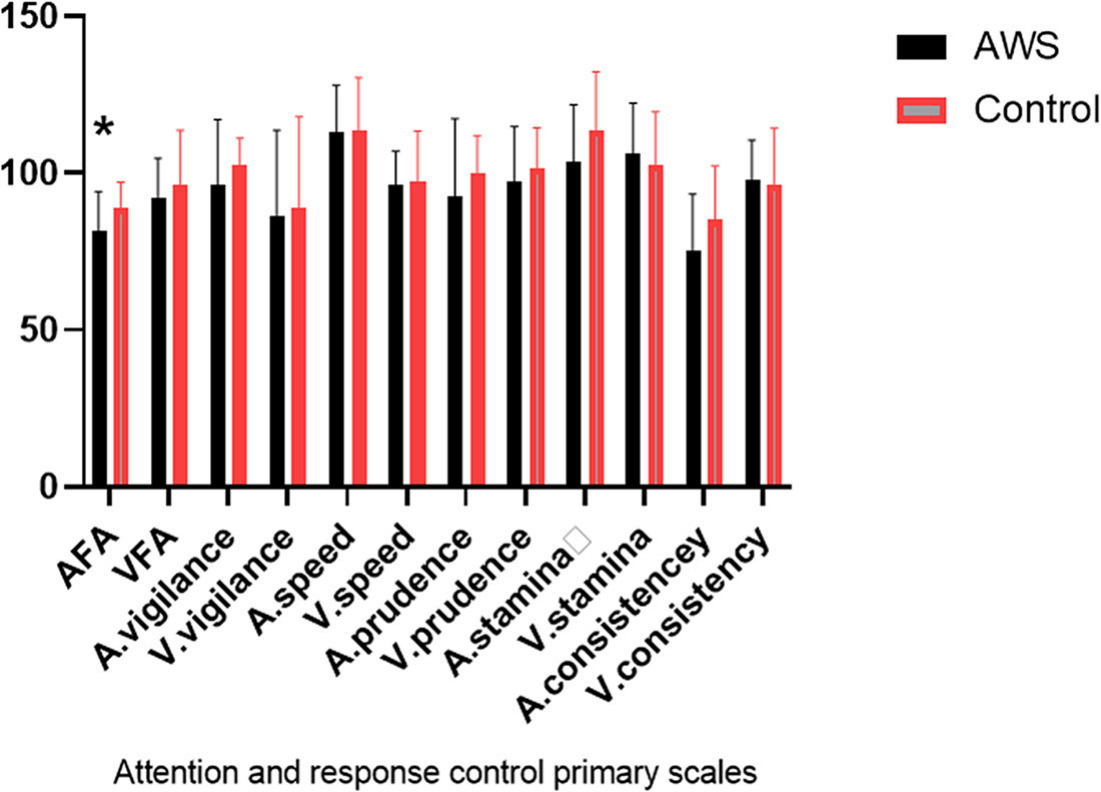
Schematic comparison of attention and response control primary scales between AWS and control group. AWS: adults who stutter, AFA: auditory focus attention, VFA: visual focus attention, A: auditory, V: visual, *p ≤ .05

**FIGURE 2:**
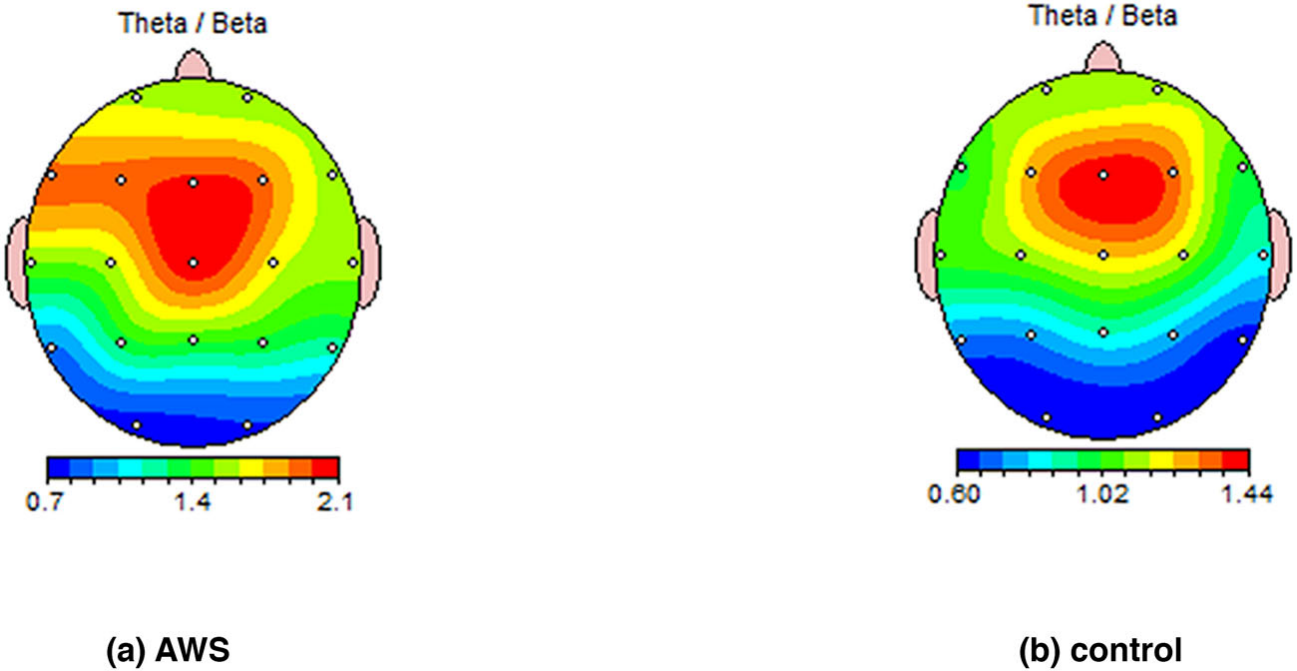
FFT theta/beta ratio group mean in AWS and control group. AWS: adults who stutter, FFT: Fast Fourier Transform

There was not a significant difference between AWS and the control group in TBR in any region of interest (ROI) after adjustment for anxiety scores (*p* ≥ .05) (Table 3). However, AWS showed smaller quotient in auditory focus attention (AFA) than control group [F (1, 48) = 5.778, *p* = .020, *ŋ* ^2^ = .107]. Other IVA2.CPT factors were similar between groups (*p* ≥ .05) (Table 4). For better illustration, the Figure 1 also shows attentional and inhibitory quotients among these two groups. Topographical maps of the absolute and relative power of the two groups' main brain waves were illustrated in Figure 2 as well.

**TABLE 3:** Comparison of TBR measures in interested ROIs in brain in resting state condition between AWS and control group

Variables	AWS	Control	*p* Value
T/BFP1	1.77 ± 0.73	1.63 ± 0.49	.44
T/BFP2	1.65 ± 0.65	1.71 ± 0.57	.75
T/BF3	1.68 ± 0.71	1.69 ± 0.60	.99
T/BFZ	1.58 ± 0.62	1.75 ± 0.49	.29
T/BF4	1.69 ± 0.64	1.70 ± 0.59	.95
T/BC3	1.62 ± 0.60	1.52 ± 0.56	.52
T/BCZ	1.49 ± 0.67	1.45 ± 0.68	.83
T/BC4	1.85 ± 0.56	1.64 ± 0.54	.17

*Note*. Data were presented as mean ± SD.

Abbreviations: ROIs: region of interests, AWS: adults who stutter, TBR or T/B: theta/beta ratio, FP: frontal pole, F: frontal, C: central, odd numbers are for the left side and even numbers are for the right side of the brain.

**TABLE 4:** Comparison of IVA.2 attention quotients (undertask condition) between AWS and control group

Variables	AWS	Control	*p* Value
AFA	81.53 ± 12.59	89.08 ± 8.16	.02*
VFA	92 ± 12.53	96 ± 17.04	.30
A. vigilance	96 ± 20.94	102 ± 8.55	.15
V. vigilance	86 ± 27.18	88 ± 29.30	.77
A. speed	113 ± 15.06	113 ± 17.13	.93
V. speed	86 ± 10.72	97 ± 16.28	.84
A. prudence	92 ± 24.88	99 ± 12.16	.18
V. prudence	97 ± 17.45	101 ± 12.66	.32
A. stamina	103 ± 18.17	113 ± 18.73	.06
V. stamina	106 ± 16.10	102 ± 16.86	.43
A. consistency	75 ± 17.87	85 ± 17.20	.06
V. consistency	97 ± 12.68	96 ± 17.76	.76

*Note*. Data were presented as mean ± SD.

* *p* < .05.

Abbreviations: AWS: adults who stutter, AFA: auditory focus attention, VFA: visual focus attention, A: auditory, V: visual.

### 3.4 Correlation IVA quotients with TBR in ROIs

There were no significant correlations between IVA attentional quotients and TBR in any ROI (*p* ≥ .05). Results have been shown in Table 5.

**TABLE 5:** Correlation between IVA2.cpt attentional quotients and TBR in ROIs

IVA quotients/ROI		FP1	FP2	F	FZ	F4	C3	CZ	C4
AFA	Correlation	0.01	.16	.03	.01	.11	.01	.01	–.09
	*p* Value	.96	.25	.82	.94	.42	.95	.96	.53
VFA	Correlation	–.04	–.09	.15	–.07	.08	.15	–.08	.13
	*p* Value	.77	.50	.28	.58	.56	.26	.54	.33
A. vigilance	Correlation	–.11	–.14	–.08	–.21	–.24	–.03	–.15	.04
	*p* Value	.40	.32	.53	.13	.08	.78	.26	.76
V. vigilance	Correlation	–.14	–.02	.25	–.01	.08	–.01	–.03	.02
	*p* Value	.13	.87	.07	.94	.57	.91	.80	.09
A. speed	Correlation	.02	–.03	–.03	–.12	–.01	–.15	–.23	.00
	*p* Value	.84	.78	.82	.39	.93	.27	.09	.97
V. speed	Correlation	.25	.21	.03	.01	.26	.03	.03	–.14
	*p* Value	.07	.14	.78	.94	.06	.83	.79	.31

Abbreviations: IVA.2.cpt: Integrated Visual and Auditory Continuous Performance Test, TBR: theta/beta ratio, ROI: region of interest.

## 4 DISCUSSION

This study aimed to compare attentional control in both resting‐state and undertask conditions between AWS and fluent speakers. Moreover, the correlation between these two conditions was considered. Our findings indicated that the anxiety level in AWS was higher than in controls. In addition, auditory focus attention was significantly lower in the AWS than in the healthy individuals.

### 4.1 Anxiety

In line with our findings that indicated the higher level of anxiety in AWS, there are several instigations in which state, trait, and social anxiety have been reported higher in people who stutter (Ezrati‐Vinacour & Levin, 2004; Iverach & Rapee, 2014). It is said that social anxiety disorder may be a debilitating experience for many people who stutter, and some features, such as fear of negative evaluation and safety behaviors, may also exacerbate stuttering (Iverach & Rapee, 2014). Moreover, anxiety may be introduced as a personality trait of people who stutter (Ezrati‐Vinacour & Levin, 2004). Anxiety is thought to compromise cognitive executive performance and causes that achievement to deteriorate. It is believed to result from the failure of prefrontal cortex control over the bottom‐up processing of information (Putman et al., 2014). It has also been shown that acute stress manipulates executive cognitive performance like working memory and divided attention. According to the attentional control theory of Eysenck et al. (2007), attentional control is ascertained by a reciprocal system in which a top‐down attention network regulates a bottom‐up, stimulus‐driven attention network. It seems that anxiety disrupts this balance and increases stimulus‐driven attention, resulting in biased attention (Putman et al., 2014). Analyzing 77 participants' frontal EEG signals and performing a stress‐induction task also showed that TBR predicted 28% of the variance in stress‐induced deterioration of attentional control. However, some findings showed that although anxiety affects attentional control adversely, compensatory strategies like enhancement of effort may cause performance effectiveness not to be impaired. Anxious individuals may also devote more executive processing resources to a task to reach optimal performance (Eysenck et al., 2007). Considering the above and a significant difference in anxiety scores between AWS and control groups, we included anxiety measures in the analysis of the attention.

### 4.2 TBR

Evaluating TBR indicated no significant difference between AWS and fluent speakers. This finding was inconsistent with a previous study reporting more power in low frequencies in ROIs in children and adults who stutter (Özge et al., 2004; Xuan et al., 2012). Another investigation speculated a similar EEG pattern for ADHD and stuttering and indicated more theta waves in AWS's frontal and prefrontal areas. Therefore, it was concluded that there is a pathological process before plan assembly in stutterers (Ratcliff‐Baird, 2002). Evaluation of quantitative electroencephalography of CWS in baseline and hyperventilation conditions also indicated increasing delta and theta waves’ frequencies and decreasing alpha and beta ones (Özge et al., 2004). However, our incompatible result may come from the heterogeneity of our case group. In fact, in line with our results, in a meta‐analytic review done in 2019, findings indicated that attentional ability might be poor in some sub‐groups of stuttering. Therefore, the attentional problem does not affect all stuttered people (Doneva, 2020). These controversies may also come from the limitations of studies like different methodologies and sample size or other uncontrollable cofactors like chronicity of stuttering.

### 4.3 IVA2.CPT

IVA2.CPT quotients evaluation indicated that AFA was significantly poorer in AWS than in the control group. The AFA quotient in IVA is the “response time to auditory stimuli” index. As our participants had no motor or mental disorder, this slower reaction time would probably be because of the weaker discrimination of the target auditory stimulus. In line with this result, in a study in which AWS was evaluated using a pure‐tone oddball paradigm, some subsets of AWS tended to perform less accurately compared to the control and were slower to respond to target auditory stimuli. In addition, the P300 mean amplitudes elicited in AWS tended to be reduced, suggesting the possibility of weaker representations of the target tone stimuli and nonlinguistic auditory processing deficits in AWS. The nature of the task may also be another reason for the lower scores of AFA in the AWS group in the present study. IVA is a rule‐switch behavioral task that needs to switch flexibly between auditory and visual modalities. These switching needs both attention and inhibitory control. Previous findings showed that people who stutter acted poorer in the complex task that needed both attentional and inhibitory control in a high‐pressure atmosphere compared with the control group (Eggers & Jansson‐Verkasalo, 2017). In that study, CWS performed a computer‐based paradigm that measured both attentional shifting and inhibitory control. CWS, compared with their fluent counterparts, react differently, with a slower speed of responses and lower accuracy rates, during task conditions requiring attentional shifting and inhibitory control simultaneously.

Moreover, our findings get support from an FMRI study that showed an attenuated connection between the auditory‐motor areas in the left hemisphere of CWS compared with the control group (Chang & Zhu, 2013). In particular, decreased connectivity was reported between the posterior superior temporal gyrus and the insula, supplementary motor area, and inferior frontal gyrus. Our finding seems to be consistent with the hypothesis that postulates atypical auditory‐motor integration in AWS (Fox et al., 1996). However, since only one attention scale between the groups was significant and critical confounding factors such as AWS' reactions to stuttering and their negative experiences were not measured, the results should be interpreted and generalized with caution.

The present result also fits with models predicting less proficient performance on attention‐demanding tasks in people who stutter, such as the Demands and Capacities model. This model suggests that stuttering is due to an imbalance between several external demands that come with speaking, such as different visual stimuli (surprised face of a listener when stuttering occurs) and the capacity to produce speech. Attention‐based imbalance probably affects linguistic processing, and error monitoring eventually leads to dysfluent speech (Doneva et al., 2018). Contrary to our prediction, we found no correlations between the attention index in the rest (TBR) and different attention quotients in IVA. We guess that our case group may be benefited from compensatory strategies like enhancement of effort or devotion of more energy resources to the task to reach the optimal performance (Eysenck et al., 2007). In the end, in general, it can be concluded that although IVA.2.CPT and TBR are applied to measure attention, they are related to the different aspects of attention, which are not necessarily correlated (Visintin et al., 2015).

## 5 CONCLUSION

Based on the present study, although AWS showed weaker auditory focused attention in undertask conditions, they showed a similar TBR attention index in resting‐state EEG comparing the control group. These results show that although attention could be considered in the assessment or even therapy of AWS, it should be examined from different aspects. Furthermore, potential EEG‐biomarker TBR does not necessarily predict attentional performance in AWS.

## LIMITATION

Like any other study, this research faced some limitations. In this study, our subject group was referred from speech therapy clinics, and they were the seekers of the therapy. Therefore, it may interfere with their levels of anxiety. Additionally, because of the restricted sample size, we did not perform a sub‐group analysis based on the gender factor. It is suggested to consider important factors, such as gender, anxiety levels, and other indexes of EEG, like main waves’ power and coherency, in future larger studies.

## DECLARATION OF COMPETING INTEREST

The authors declare that they have no financial or personal interests that could influence the work reported in this paper.

## Data Availability

The data that support the findings of this study are available from the corresponding author upon reasonable request. The data are not publicly available due to [restrictions e.g. their containing information that could compromise the privacy of research participants].
